# Systematic review of *Plasmodium knowlesi* in Indonesia: a risk of emergence in the context of capital relocation to Borneo?

**DOI:** 10.1186/s13071-022-05375-8

**Published:** 2022-07-18

**Authors:** Ibrahim Bin Said, Yobouet Ines Kouakou, Roukayatou Omorou, Anne-Lise Bienvenu, Kamruddin Ahmed, Richard Culleton, Stephane Picot

**Affiliations:** 1 Malaria Research Unit, UMR 5246 CNRS-INSA-CPE-University Lyon1, University Lyon, 69100 Villeurbanne, France; 2 Institut Agama Islam Negeri (IAIN) Kediri, Jawa Timur, 64127 Kota Kediri, Indonesia; 3 Service Pharmacie, Groupement Hospitalier Nord, Hospices Civils de Lyon, 69004 Lyon, France; 4 Faculty of Medicine and Health Sciences, Borneo Medical and Health Research Centre, Universiti Malaysia Sabah, 88400 Kota Kinabalu, Sabah Malaysia; 5 Department of Pathology and Microbiology, Faculty of Medicine and Health Sciences, Universiti Malaysia Sabah, 88400 Kota Kinabalu, Sabah, Sabah Malaysia; 6 Division of Molecular Parasitology, Proteo-Science Center, Ehime University, Matsuyama, Japan; 7 Institute of Parasitology and Medical Mycology, Hôpital de La Croix-Rousse, Hospices Civils de Lyon, 69004 Lyon, France

**Keywords:** *Plasmodium knowlesi*, Zoonotic malaria, Indonesia, Malaria elimination, Kalimantan, Borneo, *Anopheles*

## Abstract

**Background:**

The Indonesian Republic plans to relocate its capital from Jakarta to East Kalimantan, Borneo Island, in the next few years. This relocation may be associated with deforestation, decreased biodiversity, and an increased risk of emerging zoonotic infections, including *Plasmodium knowlesi* malaria. The Malaysian part of Borneo Island is one of the main hotspots of *P. knowlesi* malaria.

**Methods:**

Considering this risk, we evaluated the transmission dynamics of *P. knowlesi* in the Indonesian Archipelago based on a literature search and extensive review of data from the Indonesian Ministry of Health.

**Results:**

We report that 545 *P. knowlesi* cases were documented in Indonesia, mainly in the Aceh and North Sumatra provinces, with 95% of these occurring in the last 4 years.

**Conclusions:**

The main *P. knowlesi* vectors are present in the area of the future capital, requiring strengthened surveillance to reduce the risk of emerging cases in a rapidly growing population.

## Background

Malaria is a public health challenge in Indonesia where more than 273 million people live on 16,671 islands with limited access to modest health facilities in endemic provinces. Recently, the challenge has been exacerbated by an increase in the annual parasite incidence (API) from 0.84 in 2018 to 0.94 in 2020 [1]. A gradual increase of malaria cases from 51,418 in 2010 to 216,841 in 2020 has occurred in Papua Province (Eastern Indonesia), representing more than 85% of the national cases [1–11], whereas 33 provinces have reported declining numbers of malaria cases [1]. Among the non-zoonotic malaria cases recorded in 2019, 57% were caused by *Plasmodium falciparum*, 35% by *Plasmodium vivax*, 1% by *Plasmodium malariae*, and 7% were multispecies infections. There were very few cases of *Plasmodium ovale* [12]. In highly endemic areas (API > 5), *P. falciparum* is the predominant species. *Plasmodium vivax* is distributed in almost all islands but is dominant in the western part of Indonesia which typically experiences moderate (1 < API < 5) to low (API < 1) endemicity [12].

More than a decade ago, the Indonesian Ministry of Health (MoH) revised its malaria elimination objectives and provided a timeline for Indonesia to be free of malaria by 2030. The keystones of this plan were as follows: (1) all healthcare facilities should be able to conduct microscopic blood tests by 2010; (2) all of Indonesia's regions should have reached the pre-elimination stage by 2020; (3) malaria should be eliminated in all of Indonesia's regions by 2030 [13].

At the time this new policy was implemented, transmission of *Plasmodium knowlesi* in Indonesia was neglected. *Plasmodium knowlesi* is a non-human primate malaria parasite responsible for zoonotic malaria in Southeast Asia. It is transmitted to its major natural host, *Macaca fascicularis*, mostly by mosquitoes of the *Anopheles leucosphyrus* group, and was thought to be confined to macaque monkeys until the first report of a human infection in Malaysia on April 9, 1965 [14]. It is now responsible for the majority of malaria cases in Malaysia [15–28]. It is suspected that in neighboring countries, including Indonesia, the transmission of *P. knowlesi* is underestimated due to limited access to high-standard microscopic skills and molecular diagnosis tools [21, 29].

The relocation of the Indonesian capital may lead to increased *P. knowlesi* malaria transmission. Indonesia’s current capital is Jakarta, a megacity facing major challenges including vulnerability to natural disasters such as regular flooding, air and water pollution, overcrowding, and traffic congestion. The government decided in August 2019 to relocate its capital from Jakarta to East Kalimantan, Borneo Island. The decision was confirmed in January 2022 by the Indonesian House of Representatives. The new Indonesian capital, Nusantara, is projected to have 1.5 million inhabitants [30–32]. The establishment of this new capital and the construction of a trans-Borneo highway to Eastern Malaysia will involve deforestation of approximately 126,000 square kilometers of one of the oldest and largest tropical rainforests in Indonesia. Borneo, a hotspot of biodiversity, has already suffered huge deforestation that has endangered many species. Further decreasing biodiversity and bringing more people in closer contact with macaques and mosquitoes may dramatically increase the risk of emerging zoonotic infections, including *P. knowlesi* malaria. Indeed, Malaysian Sabah and Sarawak states, at the northern border of Indonesian Borneo, are among the main areas of *P. knowlesi* transmission to humans, representing more than 9000 cases reported between 2017 and 2019 [33]. In 2021 alone, the World Health Organization (WHO) reported 3342 cases and 13 deaths from *P. knowlesi* malaria in Malaysia. Thus, during the process of Nusantara settlement, the risk of increased *P. knowlesi* transmission to humans at the human–wildlife interface must be considered as a potential threat.

Considering the potential impact of the capital relocation on *P. knowlesi* transmission to humans, an overview of *P. knowlesi* malaria in Indonesia is required, so that it can be considered in the perspective of future challenges to reduce the transmission of both zoonotic and non-zoonotic malaria. Thus, we performed an extensive review of the published literature and data from the Indonesian MoH and national malaria program to document the transmission of *P. knowlesi* malaria in the Indonesian archipelago and the risk posed by the relocation of the capital to Borneo Island.

## Methods

### Bibliographic research

An extensive search for studies published before April 15, 2022, was performed using three databases: PubMed, Web of Science (WoS), and ScienceDirect. Keys words of interest were “*knowlesi*” and “indonesia.” Search details were as follows: "*knowlesi*" [All Fields] AND (“Indonesia” [MeSH Terms] OR “indonesia” [All Fields] OR “indonesias” [All Fields]) for PubMed; *knowlesi* (All Fields) and Indonesia (All Fields) for Web of Science; *knowlesi* AND Indonesia for ScienceDirect. No filter or date limitations were used. To increase the completeness of the search, a snowballing approach was used, and duplicates were removed. The literature research was complemented using Google Scholar. Several articles written in Bahasa Indonesia were found through Indonesian journal portals. The remaining references were assessed for eligibility based on their title, abstract, and full text. Inclusion and exclusion criteria were established a priori. All records relative to *P. knowlesi*, its hosts (human and non-human), and related malaria cases were included. Records with the following exclusion criteria were not included: *P. knowlesi* studies not related to Indonesia (cases, hosts, vectors), studies with data redundancy, experimental studies not related to *P. knowlesi* malaria cases, malaria studies involving only other *Plasmodium* species, studies not specifically related to malaria, studies written in a language other than English, French, and Bahasa Indonesia, and studies with an unavailable abstract and/or full text.

### Data management

Epidemiological, clinical, and biological data were extracted from the collected studies by four authors (IBS, YIK, RO, and SP). Information collected included first author, year of publication, number of cases, diagnosis methods when available, anti-malarial treatment if any, and geographical origins of cases included.

## Results

**Fig. 1:**
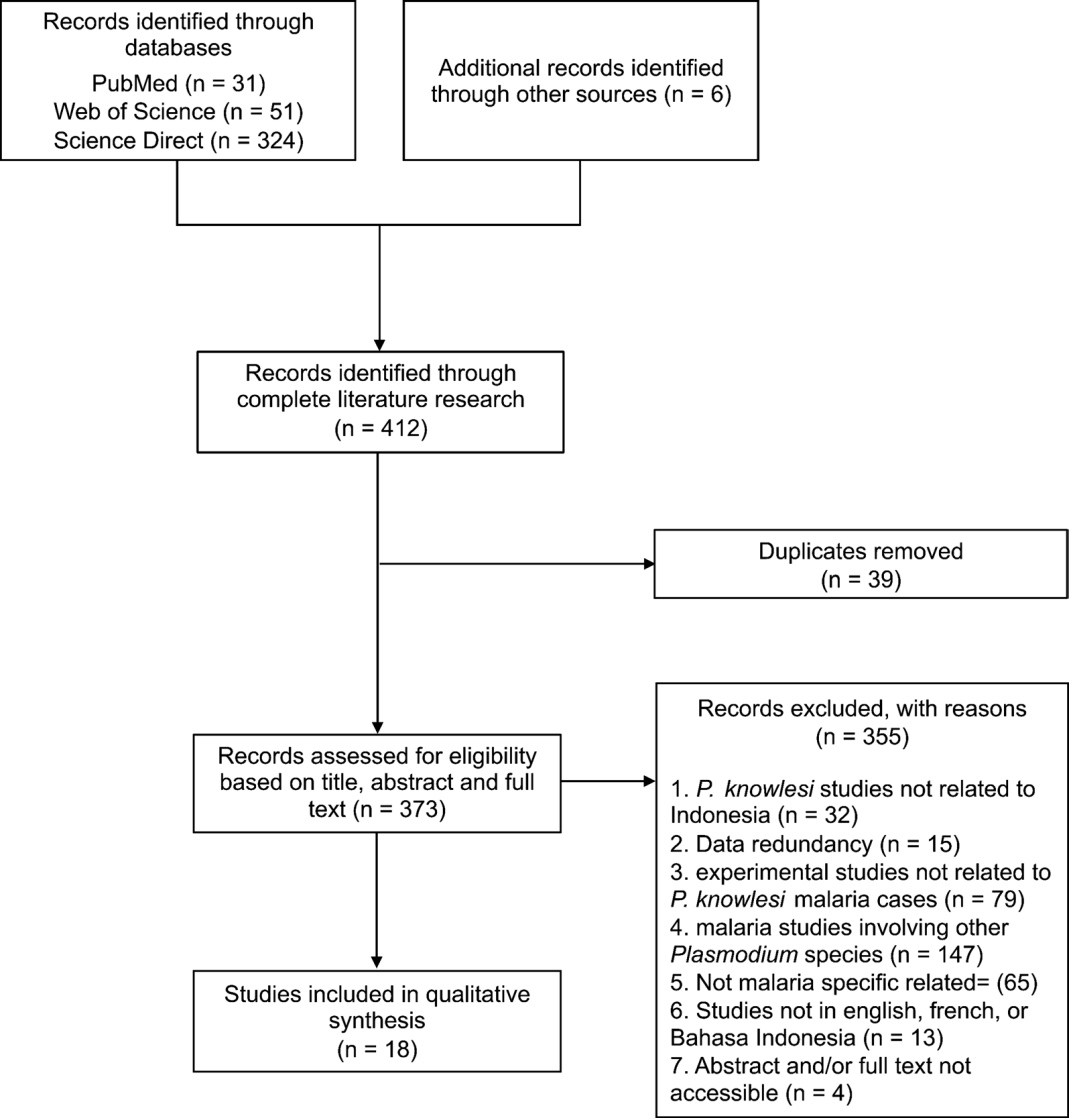
Bibliographic research flow chart

The literature search identified 412 records through four different sources: 31 from PubMed, 324 from ScienceDirect, 51 from WoS, and six from Google Scholar. After removal of the duplicates, the eligibility of the remaining studies (*n* = 373) was assessed upon their title, abstract, and full text. In total, 355 records were excluded as they did not meet the selection criteria. The reasons for exclusion were mainly non-*knowlesi* species (41%), experimental studies (22%), and non-malaria studies (18%). A total of 18 studies were included in the present qualitative study (Fig. 1).

### *Plasmodium knowlesi* transmission in Indonesia

**Fig. 2:**
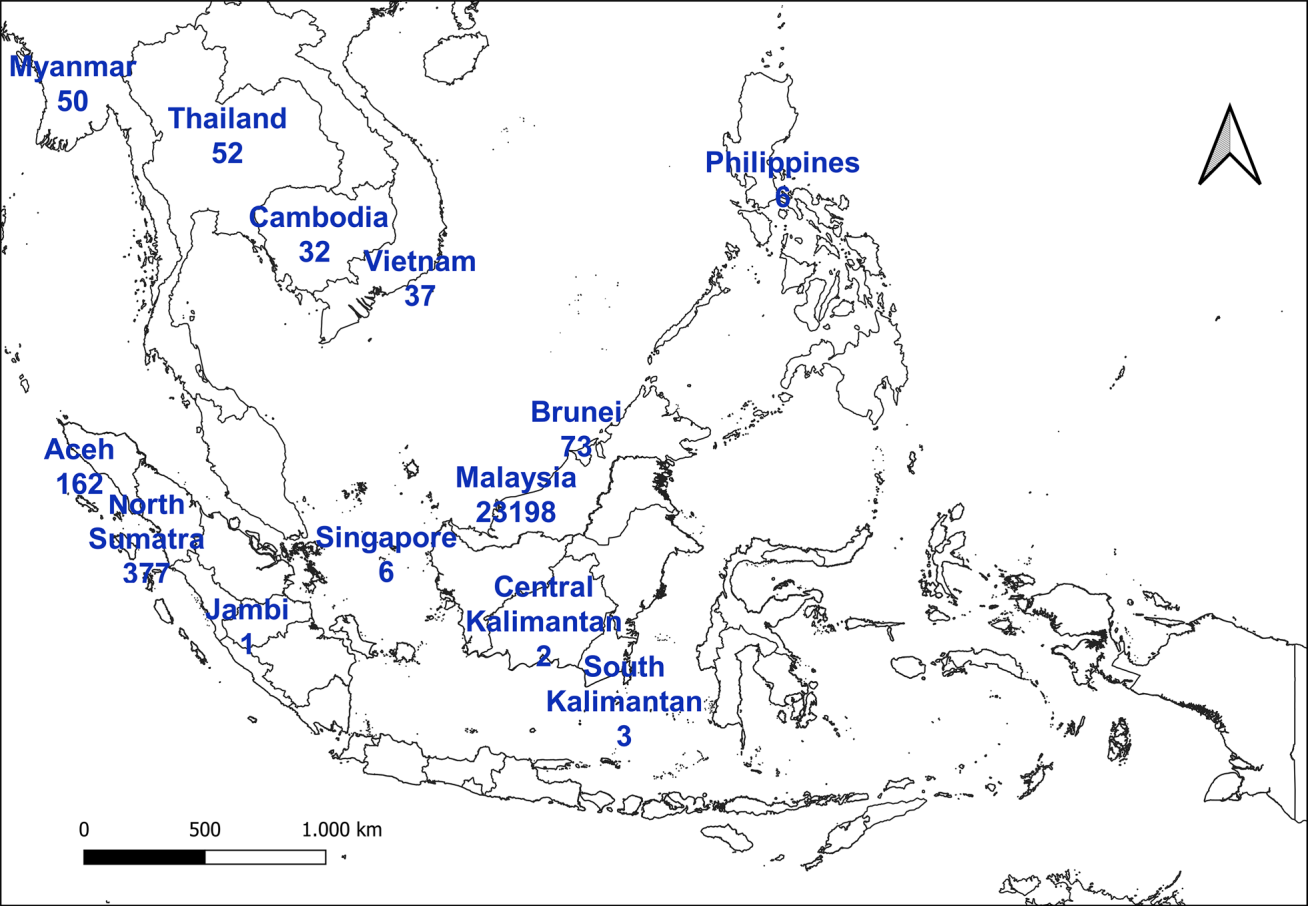
*Plasmodium knowlesi* malaria cases in Indonesia; 545 cases came from five provinces (Aceh, North Sumatra, Jambi, Central Kalimantan, and South Kalimantan) in Indonesia. Adapted from articles published in 2010–2021

We systematically reported all the documented human cases of *P. knowlesi* malaria in Indonesia from published articles. Between 2010 and 2021, a total of 545 *P. knowlesi* malaria indigenous cases were recorded in Indonesia (Fig. 2). Among these cases, 95.4% (520) were recorded between 2017 and 2021 (Table 1).

**Table 1:** *Plasmodium knowlesi* confirmed cases in Indonesia

Article [Ref]	Total number of cases (*n*)	Cases description
Figtree et al. [15]	1	1 case exported to Australia from South Kalimantan, PCR diagnosis
Sulistyaningsih et al. [16]	1	1 case in South Kalimantan, PCR diagnosis
Ompusunggu et al. [17]	3	1 case in South Kalimantan, PCR diagnosis; 2 cases in Central Kalimantan, Microscopic diagnosis confirmed by PCR
Setiadi et al. [18]	1^a^	1 case in Central Kalimantan, PCR diagnosis
Herdiana et al. [20]	20	20 cases in Aceh, PCR diagnosis
Salwati et al. [19]	1	1 case in Jambi, PCR diagnosis
Lubis et al. [22]	377	377 cases in North Sumatra, PCR diagnosis
Coutrier et al. [21]	19^b^	19 cases in Aceh, PCR diagnosis
Herdiana et al. [23]	15	15 cases in Aceh, PCR diagnosis
Zohra et al. [24]	111	111 aggregated cases 2015–2017 in Aceh
Ramadhan et al. [25]	16	16 cases in Aceh, PCR diagnosis

^a^*n* = 1 case described in [18] was defined at the same place and date as [17]

^b^*n* = 19 cases described in [21] were defined at the same place and date as [20]

The first case of *P. knowlesi* reported in 2010 from South Kalimantan Province and exported to Australia was a 39-year-old man infected after working for 18 months in close proximity to the forest [15]. In the same year, another case of *P. knowlesi* was reported from the same province [16]. Again, in Kalimantan, three (*n* = 3) cases were positive for *P. knowlesi* out of 287 malaria cases during the 2013–2014 period: a 28-year-old man and a 50-year-old man were from Central Kalimantan Province and a 28-year-old pregnant woman was from South Kalimantan Province [17, 18].

Several cases occurred in different provinces of Sumatra Island (Fig. 2). From the published literature, in Aceh province, 15 *P. knowlesi* cases occurred in 2014 [23], 20 cases during 2014–2015, and 16 cases in the 2018–2019 period [20, 21, 25]. In Jambi province, one case was collected in 2015 [19]. In North Sumatra province, 377 cases of *P. knowlesi* were collected in 2015 [22]. A total of 111 aggregated cases occurred in this Aceh province between 2015 and 2018 [24]. Aceh and North Sumatra provinces may be considered as the hotspots of Indonesian *P. knowlesi* (Fig. 2).

### Diagnosis of *P. knowlesi* malaria

The first case of natural infection of *P. knowlesi* in Indonesia, reported in 2010, was diagnosed by nested PCR [16]. *Plasmodium knowlesi* infections reported in 2015 were initially diagnosed as *P. vivax* by microscopy, before PCR analysis revealed them to be *P. knowlesi* [17]. In the same sampling area and at the same data collection time, one case of *P. knowlesi* malaria was reported in North Barito Regency, and was diagnosed by PCR [18]. In Jambi province, one of 34 cases was confirmed as *P. knowlesi* using nested PCR targeting the small subunit ribosomal RNA (SSU rRNA) gene [19]. In Aceh province, 20 positive cases of *P. knowlesi,* initially diagnosed as *P. falciparum*, *P. vivax*, or *P. malariae,* were identified by nested PCR in 2016. Two years later, 19 cases of *P. knowlesi* malaria were reported after detection by PCR in the same place and the same year of data collection [20, 21]. These cases were probably the same as those described in 2016. In 2017, 377 cases of *P. knowlesi* were identified in Batubara, Langkat, and South Nias regencies, North Sumatra province, using a conventional nested PCR and a hemi-nested PCR assay based on a conserved region of the gene encoding *P. knowlesi*—specific schizont-infected cell agglutination variant antigens (SICAvar) [22]. In 2018, 15 cases of *P. knowlesi* infection were reported in Aceh Province using a nested PCR method that targets the cytochrome b gene and Alu-I enzyme digestion, followed by confirmation using 18S rRNA nested PCR [23]. In 2018–2019, 16 cases of PCR-confirmed *P. knowlesi* malaria were reported in Aceh [25]. Of all published *P. knowlesi* malaria cases, only two were diagnosed using microscopy (Table 1), confirming the global accuracy of the case definition.

### Treatment of *P. knowlesi* malaria in Indonesia

In Indonesia, the first patient infected with *P. knowlesi* recovered following treatment with chloroquine-primaquine [16]. Several combinations of treatment have been used, including chloroquine followed by primaquine, artemisinin-based combination therapies (ACTs) (artemether/lumefantrine; artesunate/amodiaquine; dihydroartemisinin/piperaquine), and primaquine. All the above combinations of drugs used in Indonesia have led to successful outcomes for *P. knowlesi* malaria. ACTs appear to be effective against *P. knowlesi*, while few data are available yet, and no clinical and biological therapeutic efficacy studies have been prospectively conducted in Indonesia. The Indonesian MoH recommends dihydroartemisinin + piperaquine (DHP) for 3 days + primaquine (0.25 mg/kg on the first day for *P. falciparum,* and 14 days in the case of *P. vivax* and *P. ovale* infection) as the first-line treatment for uncomplicated malaria. In the case of *P. vivax* malaria relapse, primaquine levels are increased to 0.5 mg/kg per day. *Plasmodium malariae* treatment is based on DHP for 3 days with the same dose. For patients with multi-species infections (*P. falciparum* + *P. vivax or P. ovale*), treatments are applied in accordance with the guidelines for the treatment of single *P. vivax* or *P. ovale* infections. In the case of suspected *P. knowlesi* malaria, the treatment is the same as that of *P. falciparum* malaria.

### Vectors of *P. knowlesi*

**Fig. 3:**
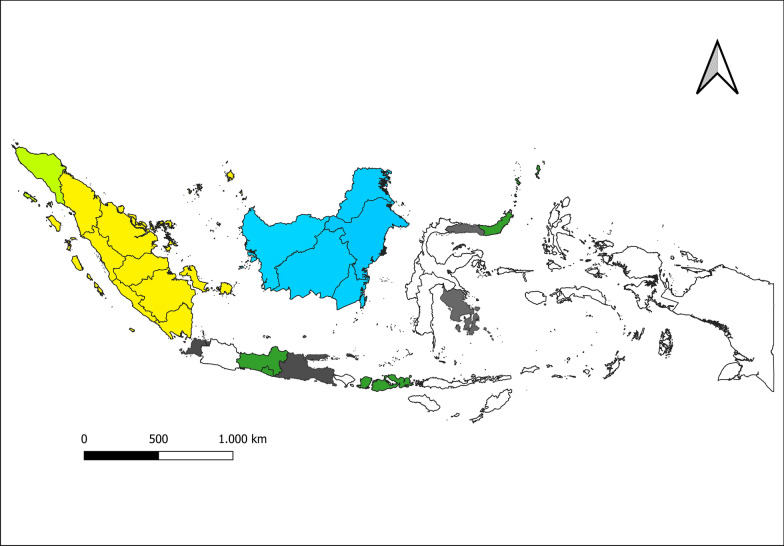
Distribution of main *P. knowlesi* vectors in Indonesia. Orange:*Anopheles cracens*, *An. introlatus*, *An. balabacensis*, and *An. leucosphyrus*. Yellow: *An. introlatus*, *An. balabacensis*, and *An. leucosphyrus*. Purple: *An. leucosphyrus*, *An. latens*, *An. balabacensis*. Green: *An. Balabacensis*. Grey: *An. leucosphyrus*

Of the known *P. knowlesi* vectors, *An. latens*, *An. balabacensis*, *An. leucosphyrus*, *An. introlatus*, and *An. cracens* are found in Indonesia (Fig. 3).

*Anopheles latens* has been found in Central, South, and North Kalimantan provinces [34, 35]. *Anopheles leucosphyrus* is located in all provinces of Sumatra and in all provinces of Kalimantan except Central Kalimantan [34, 36]. *Anopheles balabacensis* is present in North, South, and East Kalimantan, Sumatra (Aceh, Jambi, Bengkulu, Lampung), Riau islands, Java (Central and Yogyakarta), West Nusa Tenggara, and North Sulawesi [34–37], whereas *An. introlatus* is only found in the provinces of Sumatra [34, 35]. *Anopheles cracens* is present only in Aceh [35]. Of note, *P. knowlesi* DNA was detected in an *An. balabacensis* from Lampung province [37]. Data on *P. knowlesi* vectors and hosts were extracted from the studies listed in Table 2.

**Table 2:** Records describing *P. knowlesi* vectors and hosts in Indonesia

*P. knowlesi* hosts	*P. knowlesi* vectors
Ompusunggu et al. [17]	Elyazar et al. [36]
Moyes et al. [40]	Moyes et al. [40]
Salwati et al. [19]	Wibowo et al. [37]
Ekawati et al. [39]	Vythilingam et al. [35]
Lempang et al. [38]	Van de straat et al. [34]

### *Plasmodium knowlesi* macaque reservoirs in Indonesia

**Fig. 4:**
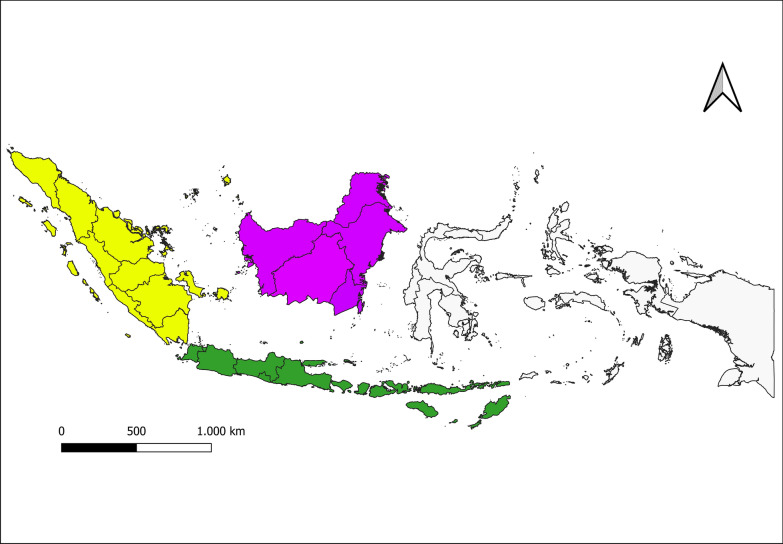
Distribution in Indonesia of monkey (*Macaca* and *Presbytis*) hosts of *P. knowlesi*. Long-tailed (*M. fascicularis*), pig-tailed (*M. nemestrina*), and banded leaf monkey (*P. melalophos*) can be found in almost all areas in Indonesia, except Sulawesi, Maluku, and Papua. Yellow: *M. fascicularis*, *M. nemestrina*, and *P. melalophos*. Purple: *M. fascicularis* and *M. nemestrina*. Green: *M. fascicularis*

Long-tailed macaques (*M. fascicularis*), pig-tailed macaques (*M. nemestrina*), and banded leaf monkeys (*Presbytis melalophos*) are the natural hosts of *P. knowlesi*. In Indonesia, *M. fascicularis* can be found in all provinces of Sumatra, Kalimantan, Java, Bali Province, West Nusa Tenggara province, and East Nusa Tenggara province (Fig. 4) [38–40], *M. nemestrina* in all provinces of Sumatra and Kalimantan [38–40], and *P. melalophos* in all provinces of Sumatra [38, 39]. A study conducted in Jambi province reported in 2017 five *M. fascicularis* and one *M. nemestrina* among 38 wild macaques infected with *P. knowlesi* [19], and a study conducted in Central Kalimantan in 2014 reported three macaques infected with *P. knowlesi* [17].

## Discussion

As early as 2009, the MoH of Indonesia implemented policies that aimed to eliminate malaria from the country by 2030. One of these policies stipulated that all health service facilities must conduct blood smear examination for the diagnosis of malaria. The Ministry of Home Affairs issued a mandate in 2010 to governors and regents/mayors throughout Indonesia regarding guidelines for the implementation of malaria elimination policies. This mandate formed the premise and the legal basis for elimination programs in several regions of Indonesia, both at the provincial and regency/city levels. The implementation of these policies was particularly targeted to areas such as the Bangka Belitung Islands, located at the east of Sumatra and north of Jakarta, where 8426 cases of malaria (all species) were reported in 2008 [13, 41]. After 2010, Jakarta, Bali, and Aceh province progressively implemented the governor's regulations. In Aceh, malaria elimination was expected to be achieved in 2015, whereas human malaria was eliminated in 2019 from 21 of 23 cities/regencies in Aceh. The same rules came into effect during the following decade at Sabang City (Aceh province) in 2012, Central Bangka Regency (Bangka Belitung Islands Province), Kendari City (Southeast Sulawesi), and North Lombok Regency (West Nusa Tenggara Province) in 2016, Ambon City (Maluku Province) in 2018, Pasangkayu Regency (West Sulawesi Province) in 2019, and Bone Bolango Regency (Gorontalo Province, Sulawesi) in 2020. Of importance, none of these policies specifically targeted *P. knowlesi* malaria.

According to our review of the literature, there were a total of 545 *P. knowlesi* malaria cases reported in Indonesia during the last 12 years. This number should be considered in comparison with the 4131 cases recorded in Malaysia during only 1 year in 2018 [42]. Of note, 95.4% of the Indonesian cases were reported during the last 4 years (2017–2021). This substantial increase in reporting could be the result of more interest in the diagnosis of *P. knowlesi* and a better detection using molecular methods, but we do not know if this is relevant to an increase in transmission to humans. Thus, there is evidence for a potential threat of *P. knowlesi* malaria in Indonesia that should be urgently addressed in the path toward malaria elimination at the country level.

Another important issue is the misdiagnosis of *P. knowlesi* species using microscopy due to its resemblance to other species, especially in the early trophozoite stage which is similar to *P. falciparum*. At the mature trophozoite, schizont, and gametocyte stages, *P. knowlesi* is difficult to distinguish from *P. malariae* [43]. Of interest, a recent meta-analysis showed that approximately 57% of *P. knowlesi* cases were misidentified by microscopy as *P. malariae* [44]. In reference to the United Nations report of malaria in Indonesia from 2010 to 2019, it is conceivable that among the 14,911 malaria cases reported as non-*P. knowlesi* species, some of them were *P. knowlesi* infections. Data compiled by WHO Indonesia showed that 465 cases of *P. knowlesi* malaria were reported in Indonesia between 2004 and 2016 [45]. Currently, there is no reliable, cheap, and easy test for *P. knowlesi* malaria. With the emergence and growing risk of zoonotic malaria, there is an urgent need for the development and implementation of new methods for surveillance of *P. knowlesi.* Such new detection tools should allow screening of the whole population to detect asymptomatic carriers of all *Plasmodium* species in order to prevent further transmission of this devastating disease.

The relocation of the capital of Indonesia to Kutai Kartanegara Regency and North Penajam Paser Regency, East Kalimantan, Borneo Island, may bring a massive increase in the human population potentially exposed to *P. knowlesi*. In 2019, these areas had low endemic status of malaria. Between January and March 2020, 600 cases of malaria infections were recorded. In 2021, two residents died from human malaria in the exact area of the new capital city. It must be remembered that in neighboring Malaysia, a survey on wild monkeys found 101 out of 108 long-tailed macaques infected with malaria parasites, and 84 of them had *P. knowlesi* mono- and multi-infection [46]. The main *P. knowlesi* vectors, *An. latens*, *An. balabacensis*, and *An. leucosphyrus*, are present in Kalimantan*.*

Human–mosquito–human *P. knowlesi* transmission has been experimentally demonstrated, and *P. knowlesi* gametocytes have been described in humans. However, while there is still limited evidence documenting human-to-human transmission, this substantial gap of knowledge should not preclude the possibility of this event based on the evidence of high transmission rates in Malaysia. A local adaptation of strains of *P. knowlesi* to new transmission pathways or changes in vector behavior in specific areas should be evaluated.

## Conclusions

This overview of *P. knowlesi* malaria contributes to the identification of issues that are needed to be addressed before 2024 when the new capital will be established in Kalimantan. Extensive surveys of the prevalence and distribution of *P. knowlesi* in Indonesia, as well as the distribution of hosts and the bionomic vectors, are urgently required. To achieve this, stronger collaboration between ministries, including the Ministries of Health, Forestry and the Environment, and Home Affairs, and WHO is required to reach the malaria elimination target by 2030, although *P. knowlesi* malaria in humans is not yet a requirement for malaria-free certification. However, it will be difficult to certify a country free of malaria if there is a risk of *P. knowlesi* malaria, even at low transmission rates. Moreover, stronger government coordination between provinces, regencies, and cities is required throughout Indonesia to anticipate the risk in this region. Tools and training for biological diagnosis of *P. knowlesi* are urgently needed in endemic areas, including in Kalimantan. Indonesia should aim to strengthen case-based surveillance and to improve the genetic epidemiology of these cases to allow a safer relocation of its capital.

## Data Availability

Not applicable.

## Abbreviations

ACTs: Artemisinin-based combination therapies
DHP: Dihydroartemisinin + piperaquine
MoH: Ministry of Health
